# The intersection of influenza infection and autoimmunity

**DOI:** 10.3389/fimmu.2025.1558386

**Published:** 2025-04-03

**Authors:** Shunyu Xie, Jintian Wei, Xiaohui Wang

**Affiliations:** Guangzhou Institute of Paediatrics, Guangzhou Women and Children’s Medical Center, Guangdong Provincial Research Center for Child Health, State Key Laboratory of Respiratory Disease, Guangzhou Medical University, Guangzhou, China

**Keywords:** influenza virus, infection, cytokine, autoimmunity, autoantibody

## Abstract

The relationship between viral infection and autoimmune manifestations has been emerging as a significant focus of study, underscoring the intricate interplay between viral infections and the immune system. Influenza infection can result in a spectrum of clinical outcomes, ranging from mild illness to severe disease, including mortality. Annual influenza vaccination remains the most effective strategy for preventing infection and its associated complications. The complications arising from acute influenza infection are attributable not only to the direct effects of the viral infection but also to the dysregulated immune response it elicits. Notably, associations between influenza and various autoimmune diseases, such as Guillain-Barré Syndrome (GBS), Type 1 Diabetes (T1D), and antiphospholipid syndrome, have been reported. While viral infections have long been recognized as potential triggers of autoimmunity, the underlying mechanisms remain to be elucidated. Here, we described the pathophysiology caused by influenza infection and the influenza-associated autoimmune manifestations. Current advances on the understanding of the underlying immune mechanisms that lead to the potential strategies were also summarized.

## 1 Introduction

Influenza viruses (IV), classified under the Orthomyxoviridae family, encompass three types—A, B, and C—that are pathogenic to humans (1). In the outbreak seasons subsequent to the 2009 H1N1 pandemic, the incidence of influenza-related hospitalizations has varied between 10 and 375 per 100,000 individuals per season, with the highest hospitalization rates observed in infants under 6 months of age (2). Influenza exerts a substantial disease burden on the pediatric population globally, characterized by elevated rates of hospitalization, morbidity, and mortality (3). Influenza infection typically manifests with upper respiratory tract symptoms, including fever, headache, cough, pharyngitis, and nasal congestion, as well as systemic symptoms such as malaise, myalgia, and muscle fatigue. Severe cases of influenza can lead to pneumonia and extrapulmonary complications, affecting the cardiovascular, nervous, musculoskeletal, and renal systems, and may result in multiple organ damage, shock, and sepsis (4). An increasing body of research has recently identified correlations between influenza infection and autoimmune diseases, such as Guillain–Barré syndrome (GBS) (5) and Type 1 diabetes (T1D) mellitus (6). Given the high incidence of influenza which can act as a trigger for autoreactivity and is implicated in the initiation of autoimmune manifestations, investigating the relationship between autoimmunity and influenza infection is of significant interest. The immune response plays a crucial role in both infectious and autoimmune diseases. Understanding the molecular mechanisms associated with influenza infection and autoimmunity could, if translated into clinical applications, expedite the development of diagnostic and therapeutic strategies. In this review, we will discuss the impact of the immune response on tissue damage induced by influenza infection, as well as the influenza-associated autoimmune diseases and various underlying mechanisms driving these autoimmune responses.

## 2 Influenza infection-induced pathogenesis

### 2.1 Symptoms and clinic presentations

**Figure 1:**
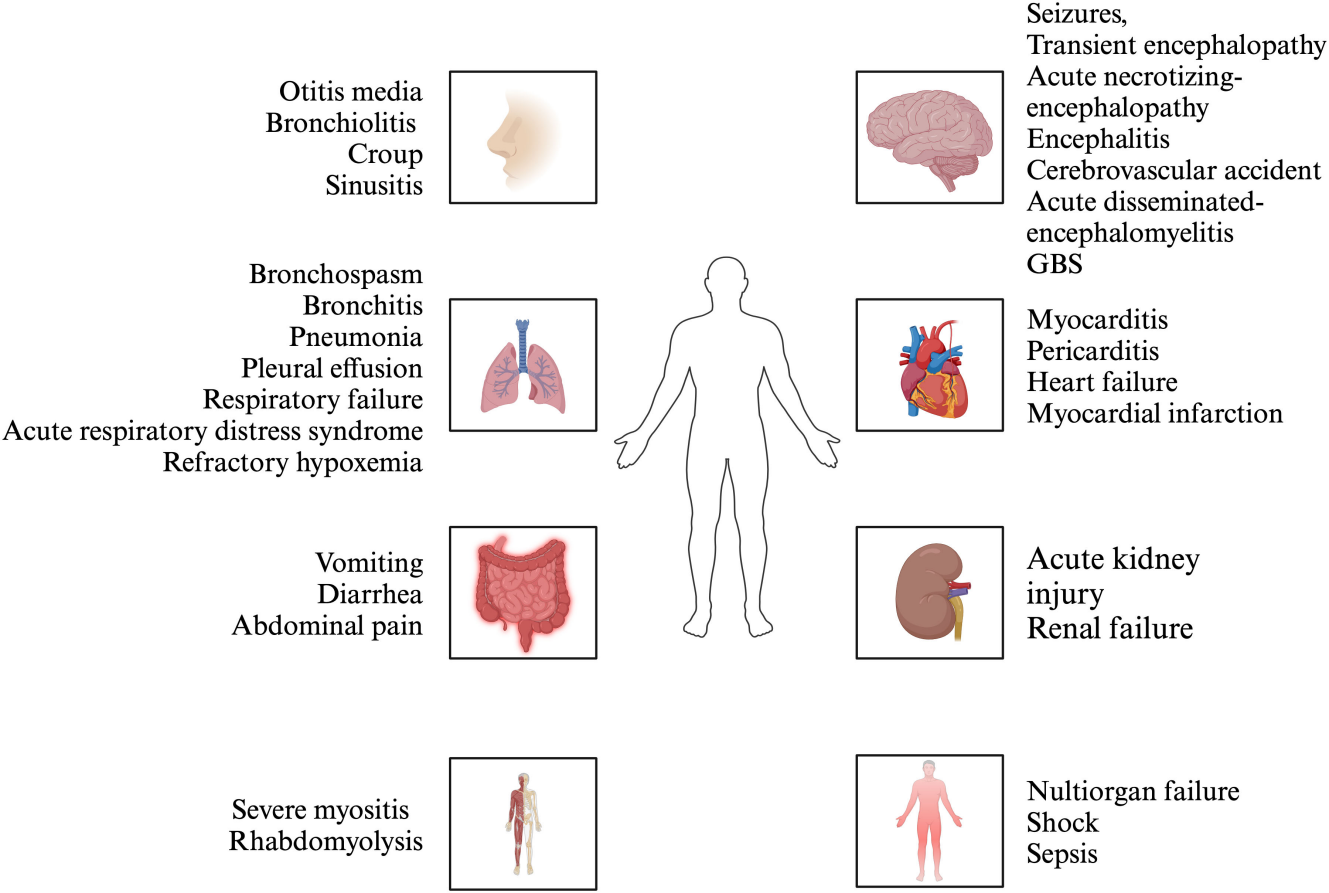
The manifestations of influenza. Influenza infection can affect multiple organs, including upper respiratory tract, lung, nervous system, cardiovascular system, digestive system, kidney, musculoskeletal system, etc. The manifestation of upper respiratory tract and lung is the predominant symptom.

Influenza is an acute respiratory viral disease caused by viruses of the Orthomyxoviridae family, with three primary types affecting humans: influenza A, B, and C. These viruses are the predominant pathogens responsible for respiratory infections, leading to epidemics and pandemics that impose significant financial burdens, morbidity, and mortality globally (1). The upper respiratory tract symptoms, fever, headache and cough, are common manifestations of influenza infection in the majority of the population, with most cases resolving completely within 7-10 days. The spectrum of disorders resulting from influenza infection varies across different age groups, from infants to the elderly. Individuals aged 65 years or older, children under 5 years, pregnant women, those who are immunocompromised (7–12) (e.g., individuals with HIV, leukemia, or those taking immunosuppressants), and persons with chronic comorbidities (e.g., asthma, heart disease, liver disease, kidney disease, obesity) are at an elevated risk for severe illnesses such as tracheobronchitis and pharyngitis (13). Additionally, a small subset of these high-risk individuals may rapidly develop serious complications, including pneumonia and acute respiratory distress syndrome (ARDS), and even death with undefined reasons (14). In addition to respiratory injuries, extrapulmonary injuries were observed, including encephalitis (15), hepatic spotty necrosis accompanied by fatty degeneration in some hepatocytes (16), and focal infiltration of lymphocytes and phagocytes in the ileum or rectum (17). A portion of hospitalized influenza patients had developed acute kidney injury (18–20). Additionally, focal myocyte injury was found in the heart (21) (**Figure 1**). Previous studies have reported that influenza infection can result in autoimmune manifestations such as elevated autoantibodies against lung surfactant proteins and brain proteins, suggesting that the implications of influenza exposure may extend beyond the immediate effects of the virus (22). Thus, understanding these links is critical for optimizing patient outcomes and managing complications arising from influenza infections, particularly among vulnerable populations such as the children and the elderly.

### 2.2 The underlying mechanisms that contribute to influenza infection-associated severe pathogenesis

#### 2.2.1 Immune hyperactivation

In addition to the factors mentioned above that cause severe influenza-associated diseases, immune hyperactivation is considered as a driving factor of severe influenza and influenza-associated diseases. A prominent histopathological feature of influenza infection show congestion, inflammation, and epithelia necrosis of larger airways with lesser extension of the inflammatory process to alveoli (23). Severe influenza infection manifests atypical pneumonia with diffuse alveolar damage accompanied by intrapulmonary hemorrhage and edema, varying degrees of inflammatory infiltration including lymphocytes, phagocytes and mononuclear inflammatory cells (24–26). Monique et al. compared lung tissues from patients of diffuse alveolar damage due to influenza and non-pulmonary causes and found more significant infiltration of natural killer cells, granzyme A^+^ CD4^+^ and CD8^+^ T lymphocytes, and CD83^+^ dendritic cells, in influenza patients (27). Patients infected with influenza virus progressing to severe disease had higher concentration of cytokines and chemokines comparing to mild patients and healthy controls, such as interleukin (IL-6), IL-15, IL-8, monocyte chemoattractant protein (MCP)-1 and tumor necrosis factor (TNF)-α (28–31). In mouse model of influenza virus infection, combination of antivirals and etanercept which impairs TNF signaling evidently alleviate clinical symptoms and improve lung pathology (32). Profiles of host chemokine and cytokine responses to infections with different strains of influenza viruses were described. Highly pathogenic H5N1 infection was prone to induce CXCL-10/IP-10,TNF-α and CCL-5/RANTES compared with avian influenza H9N2 and seasonal human influenza H1N1 (33). The production profiles of the inflammatory cytokines TNF and IL-6 are comparable between influenza A and B viruses, while IFN-γ and IL-4 levels in influenza A patients were significantly higher than those in influenza B patients (34). Chemokines and cytokines exhibit prognostic potential as predictive indicators of disease progression and clinical outcomes. In addition, complement was activated manifested by the terminal C5b-9 complement complex (TCC) formation (35) in severe H1N1 pandemic influenza infections. C4d deposition, a marker of immune complex-mediated complement activation, was found in lung sections of deceased cases (36). It is suggested that hyperactive immune response may play a fundamental role in the pathogenesis of severe influenza and could be a predisposing factor in autoimmune diseases.

Influenza viruses infect respiratory epithelial cells, triggering innate immune response *via* pathogen-associated molecular pattern recognition receptor (PAMP) recognized by host pattern recognition receptor (PRRs) (37). Viral RNA replication activates pathways such as NF-κB and IRF3, leading to the release of early cytokines like IFN-α/β and TNF-α (38). These cytokines recruit immune cells, including macrophages (39), neutrophils (40) and T cells (41), which further amplify the inflammatory cascade. However, in some individuals, these responses become uncontrolled due to genetic predispositions, comorbidities, or viral virulence factors, resulting in a cytokine storm (14). The cytokine storm is marked by elevated levels of IL-6, IFN-γ, IL-1β, and TNF-α, which promote endothelial damages and vascular leakage (42, 43). IL-6, in particular, activates the JAK-STAT3 pathway, driving further cytokine production and immune cell infiltration. Paradoxically, studies have shown that early upregulation of SOCS3 (a negative regulator of IL-6/STAT3 signaling) occurs independently of IL-6 during influenza infection. SOCS3 deficiency in murine models exacerbates cytokine storm, suggesting its role in modulating excessive inflammation (44). Additionally, IFN-γ enhance recruitment of monocytes, contributing to alveolar epithelial injury and pulmonary edema (45). Elevated circulating cytokines such as IL-6, TNF-α, and IL-1β disrupt vascular endothelial integrity across multiple organs (46–48). IL-6 activates the JAK-STAT3 pathway in endothelial cells, upregulating adhesion molecules (e.g., ICAM-1, VCAM-1) and promoting leukocyte adhesion (49). TNF-α induces apoptosis *via* caspase-8 activation and increases vascular permeability by degrading tight junction proteins (e.g., claudins, occludins) (50). This allows inflammatory cells and cytotoxic mediators to infiltrate tissues, causing microvascular leakage and ischemia. In the heart, this process contributes to myocarditis and arrhythmias (51), while in the kidneys, it exacerbates acute kidney injury through tubular epithelial cell death (52). Influenza virus infection also results in the formation of immune complexes (ICs) through the binding of viral antigens to host antibodies (53, 54). Although ICs aid in viral neutralization, their excessive deposition in extrapulmonary tissues may contribute to systemic inflammation and multi-organ damage.

#### 2.2.2 Interferons inborn error and preexisting autoantibodies

Interferons (IFNs) contribute to cell-intrinsic antiviral immunity through inducing hundreds of interferon-stimulated genes (ISGs) and enhance antiviral immune responses to facilitate viral clearance. In some patients, influenza virus could not induced sufficient type I and type III IFN responses, leading to uncontrolled influenza replication (55, 56). However, other studies demonstrated that type I interferons (Type I IFNs) also contribute to influenza virus-induced alveolar epithelial damages and lung injury *via* inducing expression of the pro-apoptotic factor tumor necrosis factor-related apoptosis-inducing ligand (TRAIL) (57). During Influenza A virus (IAV) infection, the IFN-γ is mainly derived from CD8^+^ T cells and regulates the recruitment of CCR2^+^ monocytes which mediate the lung tissue damage (58). Excessive IFN signaling induced the pathogenesis of lung during influenza virus infection (59). Some studies also suggested that IFN signaling disturbs with lung repairment after influenza infection in mice (45, 60). While type I IFNs perform a protective role in early stages of infection, further work is required to determine the roles of type I IFNs in different stages of influenza virus-mediated pathogenesis.

A series of cases about impaired type I IFNs immunity resulted from inborn error have been reported. It has been reported that patients with autosomal recessive (AR) interferon regulatory factor 7 (IRF7) deficiency suffer from life-threatening influenza pneumonia (55, 56). IRF7 is a transcription factor that is required for the production of IFNs in response to viruses (61). Plasmacytoid dendritic cells (pDCs) are the major cells of type I and III IFNs production with high levels of constitutive IRF7 expression (62). pDCs from these patients barely produced type I and III IFNs in response to IAV 24 hours post infection except IFN-β. Furthermore, dermal fibroblasts and induced pluripotent stem cell (iPSC)-derived pulmonary epithelial cells displayed increased influenza virus replication because of reduced production of type I IFNs. Inherited IRF9 deficiency manifests as severe pulmonary influenza, which depends on impaired IRF9 and ISGF3-dependent type I and type III IFN induction. The dermal fibroblasts from those patients can not sufficiently restrict influenza A virus replication (63). Giorgia Bucciol and colleagues reported that two children with AR signal transducer and activator of transcription (STAT) 2 deficiency suffered from influenza A infection developing to ARDS at 9 months of age, one of two patients died of overwhelming infection at 5 years old (64). The patients displayed hyperinflammation attributing to uncontrolled viral infection in the absence of STAT2-dependent type I and III IFN immunity. The cells from those patients and transfected with mutant STAT2 alleles conformed the phenomenon of impaired virus control (64). Three children with AR STAT1 deficiency suffered from severe influenza at 1 month and 6 months after birth was reported by Tom Le Voyer and colleagues. STAT1 is a transcription factor critical for mediation of types I, II, and III IFN responses in cells, the deficiency of the gene impairs these responses (65). Hye Kyung Lim and colleagues reported three children with inherited TLR3 deficiency developed ARDS. The pulmonary epithelial cells (PECs) and fibroblasts from these patients are susceptible to IAV attributing to lower levels of IFN-β and -λ, whereas TLR3-mutated leukocytes can produce normal level of IFNs (66). In summary, these cases demonstrate a pivotal role of intrinsic and innate type I and III IFN immunity in host defense against influenza and the associated genetic deficiency in impaired IFNs responses leading to severe influenza.

**Figure 2:**
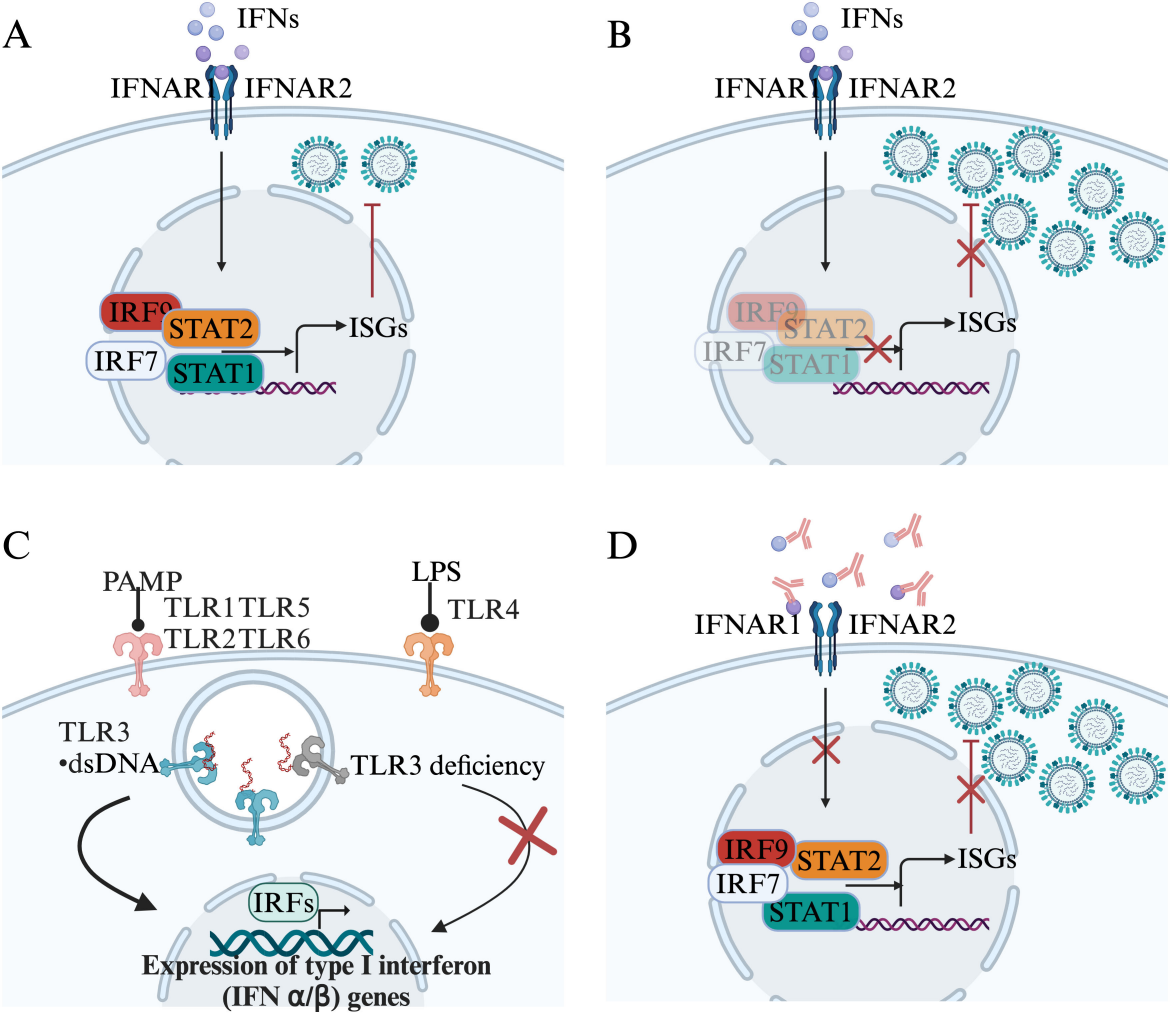
Genetics and autoantibodies associates with type I IFNs immunity disruption. **(A)**. Interferons (IFNs) contribute to cell-intrinsic antiviral immunity. **(B)**. The deficiency of IRF9, IRF7, STAT1, or STAT2 disturbs IFNs responses. **(C)**. The deficiency of TLR3 impairs the expression of IFNs. **(D)**. Autoantibodies target IFNs disturb the type I IFN response and induce uncontrol virus replication.

Interferon-neutralizing antibodies were discovered in a patient treated with human leukocyte interferon since 1981 (67). In viral diseases, neutralizing autoantibodies against type I IFN were firstly identified in a woman with disseminated shingles (68). It has long been thought that these autoantibodies have no pathological consequences until the COVID-19 pandemic. Patients with autoimmune polyendocrine syndrome type 1 (APS-1), which arises due to mutations in the autoimmune regulator gene (AIRE), exhibit antibodies against type I IFNs (IFN-α and IFN-ω) (69). The majority of patients with APS-1 infected with SARS-CoV-2 developed severe COVID-19 pneumonia, further confirmed that preexisting autoantibodies against type I IFN are related with life-threatening COVID-19 pneumonia (70). Moreover, autoantibodies against type I IFNs also contributed to live attenuated yellow fever virus vaccine associated life-threatening disease (71). In a cohort of 279 patients aged 6 to 73 years diagnosed with critical influenza pneumonia, neutralizing autoantibodies against type I IFNs were identified in 4.7% of the patients. This prevalence indicates a notable enrichment in severe influenza pneumonia compared to the general population. Individuals under the age of 70 who possess autoantibodies exhibit a heightened risk of developing severe influenza pneumonia compared to those who are negative for autoantibodies (72). The autoantibodies in patients with critical influenza pneumonia primarily targeted IFN-α2 and IFN-ω, but not IFN-β (72). Altogether, current data indicate a role of type I IFNs immune disruption in viral infection-induced tissue pathogenesis (**Figure 2**).

## 3 Autoimmune complications associated with influenza infection

Clinical and laboratory evidence indicate critical participation of immune reaction in severe cases of influenza. A study compared the concentration of inflammatory cytokines in severe and mild patients with IAV infection. IL-6, IL-10, IL-15, IP-10, IL-2R, HGF, ST2 and MIG were detected at higher levels in the plasma of severe patients (29). Daniel et al. had examined cases including severe, moderate influenza and healthy controls with bronchoalveolar lavage (BAL) samples. They reported a significantly elevated level of IL-6, IL-8, MCP-1, MIG, IP-10, IL-12, MIP-1ß and IL-1, which enhance pro-inflammatory T help 1 (Th1) immune responses (73). Moreover, total immune cells were increased in severe cases compared to moderate influenza and controls (73). They also found the elevation of CD14^+^ monocytes and plasmablasts, which indicated that autoantibodies might contribute to the lung injury. Although the number of total T cells and CD4/CD8^+^ subpopulations showed no differences from controls, the activated CD8^+^ and CD4^+^ T cells were elevated markedly in severe cases (73). Early secretion of Th17 (IL-8, IL-9, IL-17, IL-6) and Th1 cytokines (TNF-α, IL-15, IL-12p70) were detected in severe influenza patients, which were involved in cell-mediated immunity, may be associated with pathogenesis and autoimmune diseases induced by influenza (74). Hemophagocytic lymphohistiocytosis (HLH) is a syndrome characterized by severe systemic hyperinflammation presented by fevers, pancytopenia and hepatosplenomegaly (75). Hemophagocytic lymphohistiocytosis (HLH) is reported in critically ill patients with influenza virus infection (76–78). This further demonstrated that activation and proliferation of lymphocytes accompanied by excessive production of cytokines induced by influenza may lead to self-tissue damages. Taking into account the above findings regarding immune dysregulation in patients with severe influenza, the autoimmune process in the course of influenza viruses infection deserves an increasing attention.

**Figure 3:**
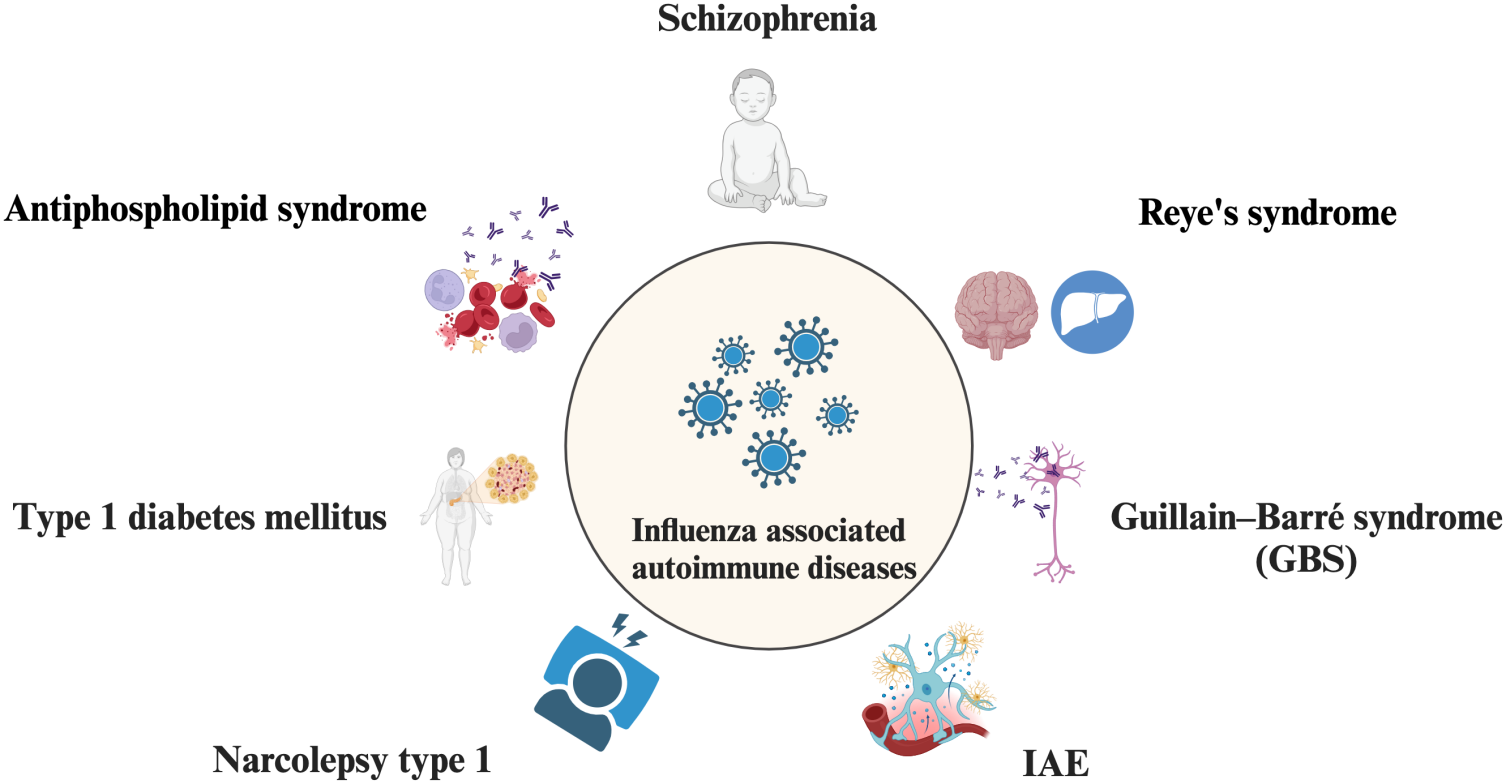
Influenza-associated autoimmune diseases. Influenza infection is involved in autoimmune disease such as Schizophrenia, Reye’s syndrome, Guillain–Barré syndrome (GBS), Influenza‐associated encephalopathy or encephalitis (IAE), Narcolepsy type 1 (NT1), Type 1 diabetes mellitus (T1D), Antiphospholipid syndrome.

Viral infections have been proposed as potential triggers of autoimmunity, and an increasing body of evidence indicates a significant association between specific viral infections and the development of various autoimmune diseases, such as type 1 diabetes mellitus with coxsackievirus (79), cytomegalovirus (CMV) (80), enteroviruses (81), as well as systemic lupus erythematosus (SLE) with hepatitis C virus (HCV) (82), CMV (83), dengue virus (84), and parvovirus B19 (85). Although with the popularity of influenza vaccination, while nearly 10% of the world’s population with all ages is still affected by influenza annually (86). Influenza-associated immune diseases may affect a huge crowd worldwide and cause a tremendous socio-economic burden. Here we review some autoimmune diseases that reported are related to influenza infection (**Figure 3**).

### 3.1 Schizophrenia

Schizophrenia is a functional psychotic disorder characterized by a spectrum of symptoms, including hallucinations, delusions, disorganized thought processes and behavior, as well as diminished cognitive and emotional capacities (87). Many studies reported the link between influenza and psychosis since 1900s. Many reports suggested that the increased risk of psychosis in offspring was associated with maternal influenza infection (88). Comparing schizophrenic patients with healthy controls, 15% of the patients had influenza antibodies in the central nervous system (89). Guglielmo et al. reported axon guidance molecules have a vast pentapeptide overlap with Influenza hemagglutinin (HA), immune cross-reactivity of axon guidance molecules and virus proteins is one potential mechanism by which influenza could contribute to autoimmunity (90).

### 3.2 Reye’s syndrome

Reye’s syndrome is distinguished by hepatic pathology and non-inflammatory encephalopathy (91). The incidence of Reye’s syndrome following influenza B was between 0.03-0.06% in population of influenza B infection. 86% cases were reported associated with the outbreak of influenza B in America (92). The recent study reported a cohort including 29,676 influenza-associated hospitalizations, which assessed the incidence of Reye’s syndrome about 0.01% in US (93). The decreased incidence might attribute to reduced use of aspirin (94). Experiments on mouse model have shown that the excessive proliferation of lymphocytes is dose-dependent on aspirin, and it is believed that Reye’s syndrome may be caused by aspirin inducing aberrant immune responses to viral infection (95). In the meanwhile, case of Reye’s syndrome caused by influenza A without the administration of aspirin has also been reported, suggesting other mechanisms leading to this disease and immune responses may contribute to the development of the disease (96).

### 3.3 Guillain–Barré syndrome

Guillain–Barré syndrome (GBS), an immune-mediated disorder impacting the peripheral nervous system and affecting approximately 100,000 individuals annually worldwide, represents the most prevalent and severe form of acute paralytic neuropathy (97). The clinical manifestations of GBS are heterogeneous, and several different clinical variants exist, but patients with GBS typically present with weakness and sensory symptoms in the legs, progressing to the arm and head muscles (98).Valérie et al. reported a cohort of 405 patients, 234 cases of which caused by an unidentified agent had a positive association with influenza-like illnesses (99). Some reports yielded a similar conclusion that a positive correlation of hospitalization rate was found between influenza and GBS (100, 101). The recent study manifested that 53% patients of GBS were confirmed recent infection by serology, of which influenza viruses accounting for 33% (102). Masaki et al. investigated 63 patients of Guillain-Barré syndrome (GBS)-related diseases (GBSRDs) after influenza virus infection, detecting anti-GQ1b and anti-GT1a which are grouped to anti-glycolipid antibodies accounting for 24% of cases (103). Debprasad et al. reported that the levels of antibodies targeting GM1, GM2, GD1a, GD1b, GT1b, and GQ1b were significantly elevated in patients with GBS compared to healthy controls, and these elevated antibody levels were correlated with immunoreactivities against influenza viruses (104). Influenza infection may be a trigger for GBS, mediated by immune responses. The underlying mechanisms between GBS and influenza will require further studies.

### 3.4 Influenza-associated encephalopathy

Influenza‐associated encephalopathy or encephalitis (IAE) is a disorder characterized by consciousness disturbance with a few days after influenza infection (105). Its clinical characteristics are rapid progressive brain damage after viral infection, and pathologically by cerebral edema without direct invasion of viruses and inflammatory cells (106). The majority of IAE cases are reported among children worldwide. Hideo et al. reported that during 2010-2015 in Japan, the incidence of IAE among children and adults was 2.83 and 0.19 cases per 100,0000 population, respectively, whereas the morality was higher in adults (107). Pierre et al. reported a cohort of 41 children admitted for influenza-associated encephalopathy between 2010 and 2019 in France, of which 17% patients died in hospital, 49% had neurologic sequelae and 27% had severe disabilities according to modified Rankin Score (108). Ayukawa et al. reported that in patients with IAE, there was a significant increase in CTLA-4^+^ CD4^+^ T cells compared to influenza patients without encephalopathy. This increase was correlated with the down-regulation of antigen-activated immune responses, suggesting that CTLA-4^+^ CD4^+^ T cells may play a role in the pathogenesis of IAE (109). Shunji et al. reported that the cytokines of IL-6, TNF-α, and IL-10 were higher in patients with 2009 pandemic H1N1 influenza-associated encephalopathy than those without neurological sequelae (110). All of the above indicated that immune responses might participate in the progression of IAE.

### 3.5 Narcolepsy type 1

Narcolepsy type 1 (NT1) is a chronic sleep disorder characterized by the degeneration of a specific subset of hypothalamic neurons responsible for the production of hypocretin (HCRT; also referred to as orexin, peptides) that promote wakefulness (111). Narcolepsy is highly correlated with H1N1 influenza independent of H1N1 vaccination (112). The estimated incidence was at least 10 per 100,000 individuals per year (113). NT1 is considered as autoimmunity associated with human leukocyte antigen (HLA) DQB1*06:02/DQA1*01:02 heterodimer (DQ0602). Guo et al. proposed a mechanism of molecular mimicry with influenza antigens modulated by genetic components in the pathogenesis of NT1 (114). T cells specific to tribbles homologue 2, an additional self-antigen of hypocretin neurons, were identified in patients with narcolepsy, thereby reinforcing the autoimmune etiology of the disorder (115).

### 3.6 Type 1 diabetes

Type 1 diabetes mellitus (T1D) is a chronic autoimmune disorder marked by insulin deficiency and hyperglycemia, resulting from the destruction of pancreatic β cells (116). Paz et al. reported that a twofold higher risk of subsequent T1D was detected in the group of pandemic influenza A infection (117). Yuichi et al. also reported the similar conception that the risk of T1D increased after influenza in the Japanese population-based cohort (118). Lijun et al. found a cross-reactive antibody between H1N1 influenza virus HA and human pancreatic tissue, suggesting the immune-mediated tissue damage caused by influenza (119).

### 3.7 Antiphospholipid syndrome

Antiphospholipid syndrome is an autoimmune disorder characterized by the presence of pathogenic autoantibodies that target cell surface phospholipids and phospholipid-binding proteins (120). Melonie et al. reported a case of catastrophic antiphospholipid syndrome (CAPS), suggesting a link between CAPS and influenza (121). Further studies are required to elucidate the underlying mechanisms of autoimmunity induced by influenza.

## 4 The immune mechanisms of viral infection-associated autoimmunity

**Figure 4:**
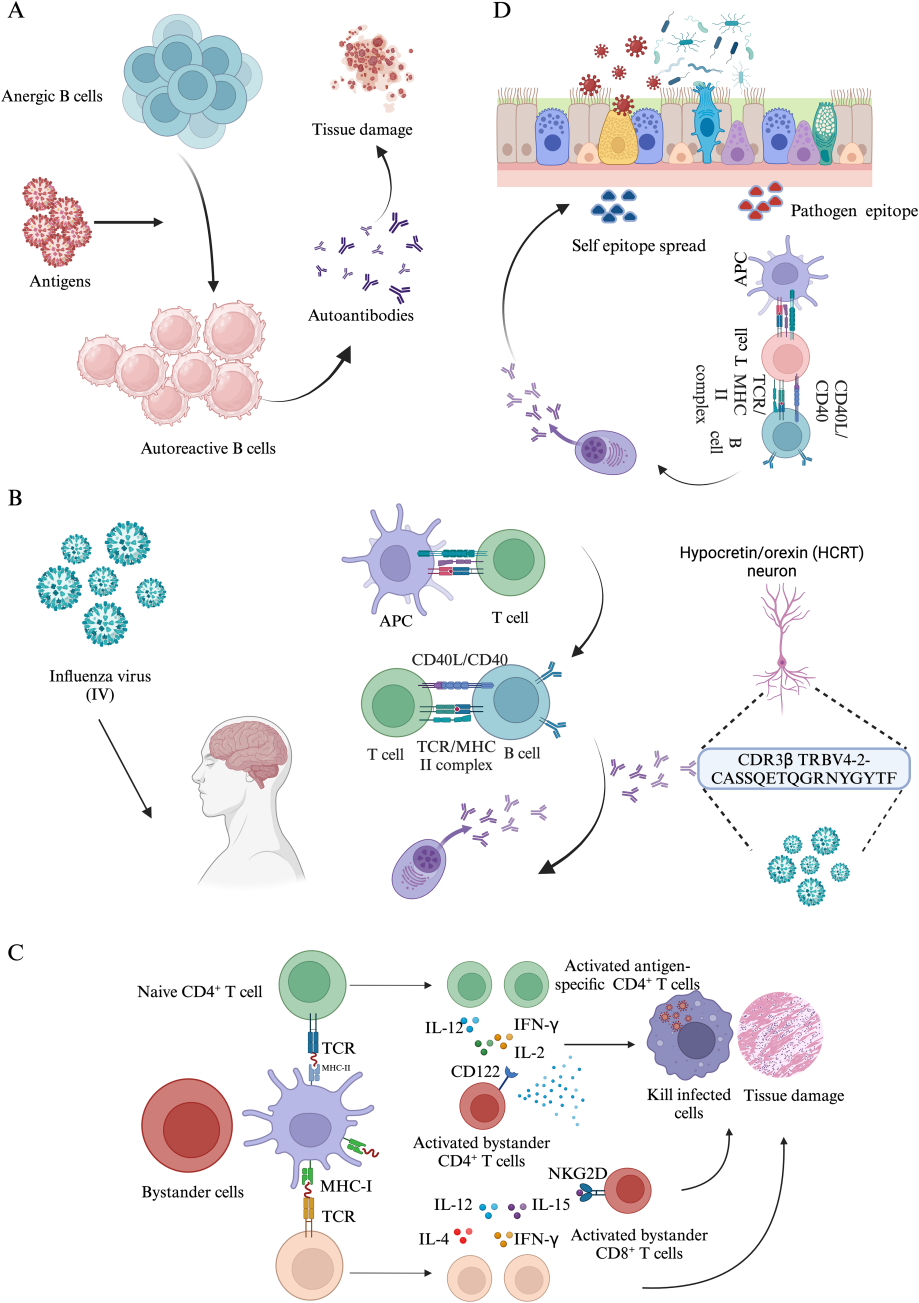
The mechanisms of autoimmune response induced by pathogen infections. **(A)**. Anergic B cells reversion. **(B)**. The molecular mimicry between Influenza virus (IV) and Narcolepsy type 1 (NT1). **(C)**. Bystander activation mechanisms induced by pathogen infections. **(D)**. During infections, self-epitope spreading distinct from the epitopes of initial pathogen contributes to tissue damages.

Autoimmune responses arise when the immune system erroneously targets and damages healthy cells (122). These conditions are believed to stem from a multifaceted interaction of genetic susceptibilities, environmental influences, and dysregulated immune responses (123). Current research suggests that autoimmune disorders impact approximately 10% of the global population, encompassing over 80 identified types, such as rheumatoid arthritis, type 1 diabetes, and multiple sclerosis (124). Notably, autoimmune diseases tend to co-occur more frequently than would be expected by chance, indicating that some autoimmune diseases may share common risk factors. This co-occurrence was particularly evident among rheumatic and endocrine diseases (123). As mentioned above, viral infections are related to the onset or development of some autoimmune diseases. Here, we explore a few possible mechanisms of infection associated autoimmune diseases (**Figure 4**).

### 4.1 Anergy reversion of B cells

Autoantibodies are considered as the most prominent immunological manifestation of autoimmune diseases, which serve as biomarker for diagnosis, classification and the progression of disease (125). Allan et al. detected autoantibodies against antigens such as IL-6, IL-7, IL-12p70, and IL-22 in 48% of hospitalized patients with influenza, whereas none were found in the healthy control group. Furthermore, antibodies linked to rare connective tissue diseases (CTDs) were prevalent in 25% of the influenza patients in America (126). Autoantibodies were prevalent in SARS-CoV-2 patients with acute respiratory distress syndrome as well as non-SARS-CoV-2 infections of patients with severe pneumonia (127, 128). Moreover, the presence of autoantibodies in patients with influenza and COVID-19 has been correlated with the severity of these diseases. These autoantibodies specifically target Type I IFN, granulocyte-macrophage colony-stimulating factor (GM-CSF), and IL-6 (127, 129). B cells play an essential role in host defense by secreting antibodies and presenting antigens from foreign microorganisms to T cells (130). Depletion of B cells tends to be favorable in autoimmune diseases such as systemic lupus erythematosus (SLE) (131) and rheumatoid arthritis (132), probably through reduced production of autoantibodies as well as antigen presentation to autoreactive T cells. After central tolerance mediated clonal deletion, 15%-20% of mature B cells have a capacity reactive with self-antigens, especially in newly formed B cells, of which the proportion up to 50% (133). In healthy individuals, the underlying autoreactive B cells are functionally silenced by the mechanism of anergy. However, the anergic state could be reversible. In pathological settings, the anergic B cells reactivate and produce autoantibodies against host tissue such as dsDNA and ssDNA. Noorchashm et al. reported that the anergic B cells are partially reversible upon stimulation of IL-4 and CD40 ligands derived from T cells associated factors (134). Peter et al. reached a similar conclusion that the anergic state of B cells have the potential to transfer to autoantibodies-secreting plasma cells and contribute to autoimmunity (135). Altogether, anergy reversion contributes to the transfer of anergy B cells to auto-reactive B cells, which secrete autoantibodies to damage tissues. Anergy reversion of B cells contributes but not sufficient to explain viral infections implicated in the development or exacerbation of autoimmune disease (136). Further studies may provide new insights in understanding the role of B cells during the development of infection-induced autoimmunity.

### 4.2 Molecular mimicry

The term “molecular mimicry” was formally introduced by Damian in 1964 to describe the phenomenon wherein infectious organism express antigens that are structurally similar to those of their host. This similarity may confer an advantage to the microbes by enabling them to evade the host’s immune response (137). Molecular mimicry occurs when viral antigens share structural similarities with host-antigens, leading to the cross-reactive immune response. This phenomenon has been noted in cases involving varicella zoster virus (VZV) (138) and Epstein-Barr virus (EBV) (139), which were thought to play roles in the pathogenesis of diseases like multiple sclerosis. There are four types of molecular mimicry that have been reported (140–143). Type 1 molecular mimicry: “microorganisms and their hosts have complete protein identity” (e.g., in chronic graft-versus-host disease, CMV hijacks CD13 and presents it to antigen-presenting cells to trigger autoimmune responses against the autoantigen); Type 2 molecular mimicry: “molecular homology between microorganisms and their hosts, based on a protein encoded by the bacteria” (e.g., human carbonic anhydrase II and alpha-carbonic anhydrase of Helicobacter pylori share a homologous binding motif with the HLA molecule DRB1*0405, which is linked to autoimmune pancreatitis.); Type3 molecular mimicry: microorganisms or environmental agents that share similar amino acid sequences or epitopes with its host; Type 4 molecular mimicry: “microbes or environmental agents have structural similarities to their hosts”. Among them, type 3 is the most prevalent type in eliciting autoimmune responses. However, molecular mimicry mediated autoimmunity is rare and complex, which is not sufficient to breach host immune tolerance mechanism (144), suggesting other factors are involved in the autoimmunity.

### 4.3 Bystander activation

Bystander activation was first described by Tough et al. in 1996. They found that a massive expansion of T cells after viral infection was mediated by cytokines other than T cell receptor (TCR) and type I IFN was a robust inducer (145). Zarozinski et al. and Murali-Krishna et al. also found that the majority of clonally expanded T cells were virus-specific but of which include a small proportion of non-virus-specific clones (146–148). Memory CD4^+^ and CD8^+^ T could be bystander activated and proliferate in IFN-γ or IL-12-dependent but TCR-independent manner (149). The bystander activation of naïve CD8^+^ T also occurs during the early phase of infection and displays an innate anti-viral feature (150). The bystander activation of naïve CD4^+^ T also could be induced by high-dose IL-2 but independent on TCR activation (151). However, bystander activation is a double-edged sword. Martin et al. reported that bystander CD8^+^ T cells were activated by inflammatory cytokines following infection and provided protective roles in host defenses (152). Preexisting non-specific memory CD8^+^ T cells are activated rapidly and display cytotoxic features following infections, which target infectious cells and contribute to pathogen clearance in an NKG2D-dependent manner (153). Rolot et al. made a similar finding that IL-4 rapidly expands non-specific CD8^+^ T cells, which are essential to the activation of antigen-specific CD8^+^ T cells for controlling virus load (154). On another hand, Bergamaschi et al. reported that hospitalized individuals of COVID-19 manifested delayed bystander CD8^+^ T cells activation which might drive lung pathology, suggesting a role of immunopathogenesis in severe pneumonia (155). Zhang et al. found that high level of IL-15 drove bystander activation of CD8^+^ T cells through NKG2D, which mediated endothelium injury in the Hantana virus (HTNV) infection model (156). Ohya et al. analyzed 23 autopsy specimens come from patients of diffuse alveolar damage, in which large amount of non-antigen-activated bystander CD8^+^ T cells were observed. These CD8^+^ T cells expressed unique granzyme B marker and might involve in the tissue injury in the progression of diffuse alveolar damage (157). Lee et al. reported bystander-activated CD4^+^ T cells also contributed to the progression of autoimmune diseases (158). Bystander activation of T cells plays an essential role in antigens clearance and also causes tissues injuries. Further studies are required to elucidate molecular mechanisms involved in different immune responses to onset and/or exacerbations of infectious diseases.

### 4.4 Epitope spreading

Epitope spreading comprises intramolecular and intermolecular spreading. Intramolecular spreading refers to the phenomenon where an immune response extends from the initially targeted epitope to additional epitopes within the same molecule. In contrast, intermolecular spreading describes the process by which an immune response broadens to include epitopes on a different antigenic molecule (159). The etiology of autoimmune diseases remains incompletely elucidated, with numerous factors influencing the development of autoimmunity. The involvement of the epitope spreading mechanism is implicated in human diseases such as autoimmune hepatitis and primary biliary cholangitis (160, 161). This study provides evidence that viral infections can induce autoimmune diseases through epitope spreading. Specifically, chronic infection with Theiler’s murine encephalomyelitis virus (TMEV) in mice resulted in demyelination, which was initiated by a TMEV-specific CD4^+^ T cell response. The subsequent T-cell response to multiple myelin autoepitopes was secondary to the virus-specific T cell response and arose from epitope spreading rather than molecular mimicry (162). Epitope spreading following viral infection may play an important role in the development of autoimmune pathogenesis.

## 5 Conclusions and future perspectives

Influenza exerts a significant impact on global health through its annual epidemics and occasional pandemics. Autoimmune pathogenesis, characterized as chronic and disabling conditions, impose a considerable burden on individuals, families, and society. While infections have frequently been implicated in the development of autoimmune diseases, they are not sufficient on their own to trigger these conditions. Factors such as genetic predispositions, defects in the innate and adaptive immune systems, and gender are known to increase the risk of autoimmune diseases. Both influenza and other viral infections such as COVID-19 have been reported to be associated with autoimmune responses and the onset of autoimmune diseases. The underlying mechanisms remain to be elucidated. Further clinical and basic investigations are necessary to uncover the mechanisms underlying autoimmune diseases following viral infections. Such research will be crucial for the prevention of post-infection autoimmunity and may offer insights for the development of immune cell-based therapeutic strategies for the treatment of autoimmune diseases.
